# Losartan-Associated Sub-fulminant Hepatitis: A Case Report

**DOI:** 10.7759/cureus.112160

**Published:** 2026-07-06

**Authors:** Kanwarpreet Sadhu, Rakshand Shetty, Prachi Mehmi, Lipi Patel, Zunera Shaikh, Rithvika Badugu, Ogundele Tosin Arinola, Ballu R

**Affiliations:** 1 Medicine, All India Institute of Medical Sciences, Bathinda, IND; 2 Internal Medicine and Radiology, SDM College of Medicine, Udupi, IND; 3 Internal Medicine, Adesh Institute of Medical Sciences and Research, Jalandhar, IND; 4 Research, Gujarat Medical Education and Research Society (GMERS) Medical College and Hospital, Gujrat, IND; 5 Gastroenterology, Isra University, Hyderabad, PAK; 6 Internal Medicine, Gandhi Medical College, Secunderabad, Hyderabad, IND; 7 Medicine, College of Medicine, University of Ibadan, Ibadan, NGA; 8 Neurology, University of Florida, Gainesville, USA

**Keywords:** angiotensin ii receptor blocker, hepatotoxicity, liver injury, losartan, sub-fulminant hepatitis

## Abstract

Losartan, a commonly prescribed angiotensin II receptor blocker, is generally well-tolerated but can rarely cause severe drug-induced liver injury (DILI), leading to acute liver failure. We report a 43-year-old man with hypertension and type 2 diabetes who developed jaundice, fatigue, and right upper quadrant pain six weeks after starting losartan (50 mg daily). Despite prompt discontinuation of the drug, he progressed to acute liver failure with hepatic encephalopathy, coagulopathy (international normalized ratio (INR): 2.8), and a Model for End-Stage Liver Disease (MELD) score of 28, meeting King’s College criteria for poor prognosis. Comprehensive workup, including viral hepatitis panel (A, B, C, and E), cytomegalovirus (CMV), Epstein-Barr virus (EBV), herpes simplex virus (HSV), autoimmune markers, Wilson disease screening, acetaminophen levels, and exclusion of ischemic and metabolic causes, was negative. Liver biopsy showed severe centrilobular necrosis, eosinophilic infiltrate, portal inflammation, interface hepatitis, and cholestasis. Causality assessment using the Roussel Uclaf Causality Assessment Method (RUCAM) yielded a score of 8 (Probable DILI). The patient ultimately required liver transplantation despite early drug withdrawal. This case highlights that losartan-induced hepatotoxicity, although rare, can be life-threatening and underscores the importance of early recognition and a high index of suspicion in patients with unexplained liver injury.

## Introduction

Losartan, the first angiotensin II type 1 (AT1) receptor antagonist approved by the US Food and Drug Administration (FDA) in 1995, is widely used for the management of hypertension, heart failure, and diabetic nephropathy [1]. It exerts its effects by selectively blocking the AT1 receptor, thereby reducing vasoconstriction, aldosterone secretion, and sodium retention [2]. While generally well tolerated, rare but serious adverse effects, including drug-induced liver injury (DILI), have been reported [3].

DILI accounts for approximately 10% of acute liver failure cases in developed countries and can result from direct toxicity, idiosyncratic immune-mediated reactions, or oxidative stress [4]. Losartan-associated hepatotoxicity is uncommon, with clinical manifestations typically appearing weeks to months after initiation. These may range from mild transaminase elevation to severe injury progressing to sub-fulminant hepatitis and acute liver failure [3,5]. Symptoms such as jaundice, fatigue, nausea, and abdominal pain often mimic viral or autoimmune hepatitis, posing diagnostic challenges [4].

This case report describes a rare instance of losartan-induced severe hepatic injury leading to sub-fulminant hepatitis requiring liver transplantation. It highlights the importance of early recognition, provides a detailed diagnostic workup, and reviews the existing literature on presentation, mechanisms, and management of this uncommon but potentially life-threatening adverse effect.

## Case presentation

We present a 43-year-old man with a history of hypertension and type 2 diabetes mellitus who presented to the emergency department with jaundice, fatigue, and right upper quadrant abdominal pain. He had been started on losartan (50 mg daily) for blood pressure control approximately six weeks prior to the onset of symptoms. His diabetes was managed with metformin and glipizide, with a recent HbA1c of 7.5%, indicating suboptimal glycemic control. He had no history of significant alcohol consumption, herbal supplement use, or recent changes in other medications.

The patient had been diagnosed with hypertension several years earlier and type 2 diabetes mellitus approximately four years prior. On the day of presentation, his blood pressure was 110/64 mm Hg. He was febrile (100.1°F), with a respiratory rate of 17 breaths per minute. Physical examination revealed icteric sclera, hepatomegaly, and tenderness in the right upper quadrant.

On presentation, the patient had markedly elevated liver enzymes: alanine aminotransferase (ALT), 940 U/L (normal: 7-56 U/L); aspartate aminotransferase (AST), 1,240 U/L (normal: 13-40 U/L); alkaline phosphatase (ALP), 320 U/L (normal: 44-147 U/L); and gamma-glutamyl transferase (GGT), 285 U/L (normal: 9-48 U/L). Total bilirubin was 12.8 mg/dL with a direct bilirubin of 9.2 mg/dL. Coagulation studies showed an elevated international normalized ratio (INR) of 2.8 and prothrombin time (PT) of 32 seconds. Serum albumin was low at 2.6 g/dL. Renal function was impaired with a creatinine of 1.8 mg/dL. Complete blood count revealed thrombocytopenia (platelet count: 85,000/μL). The calculated Model for End-Stage Liver Disease (MELD) score was 28, and the Child-Pugh score was class C (11 points), indicating decompensated liver failure.

**Figure 1:**
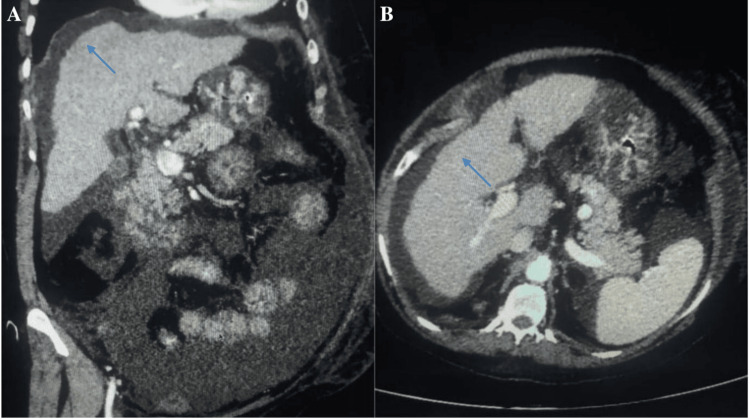
CT of the abdomen transverse (A) and axial (B) sections showing hepatomegaly, heterogeneous liver parenchyma, and features of acute hepatitis (arrows)

CT of the abdomen demonstrated hepatomegaly with heterogeneous enhancement, periportal edema, and a nodular liver surface, suggestive of severe acute hepatitis (Figure 1).

The patient reported right upper quadrant pain for approximately 3-5 days prior to presentation. A liver biopsy was performed, revealing severe centrilobular hepatocellular necrosis, mixed inflammatory infiltrate with eosinophils, and cholestasis, consistent with a drug-induced liver injury (DILI) pattern. Losartan was discontinued immediately upon suspicion of drug-induced hepatotoxicity. Despite prompt discontinuation and supportive care, the patient’s liver function continued to deteriorate rapidly over the following week, with development of coagulopathy and encephalopathy. He was transferred to a liver transplant center and successfully underwent liver transplantation.

To establish causality, the Roussel Uclaf Causality Assessment Method (RUCAM) was applied, yielding a score of 8 (Probable DILI). The WHO-UMC system classified it as “Probable/Likely,” and the Naranjo scale gave a score of 6 (“Probable”).

## Discussion

Losartan, an angiotensin II type 1 (AT1) receptor antagonist, was first approved by the US Food and Drug Administration in 1995 for the treatment of hypertension [3]. Its therapeutic uses have since expanded to include reducing the risk of stroke in patients with hypertension and left ventricular hypertrophy, as well as slowing the progression of diabetic nephropathy by decreasing proteinuria [3]. Extensive clinical research supports its efficacy in these indications. Additionally, losartan exhibits mild uricosuric properties, making it a preferred antihypertensive agent in individuals suffering from gout.

Although losartan and other angiotensin receptor blockers (ARBs) are generally considered to have a strong safety profile, emerging reports have linked losartan to rare instances of liver toxicity. These adverse effects can range from minor, asymptomatic increases in hepatic enzymes to severe forms of hepatocellular injury and, on rare occasions, sub-fulminant hepatitis. The precise pathophysiology of losartan-induced hepatotoxicity remains uncertain, although it is thought to involve idiosyncratic and possibly immune-mediated responses.

The clinical features of drug-induced liver injury (DILI) often resemble those of acute viral or autoimmune hepatitis [6]. Patients may present with non-specific symptoms such as fatigue, anorexia, jaundice, nausea, vomiting, abdominal pain, and low-grade fever. Laboratory evaluation typically reveals elevated transaminases [7,8]. DILI can present as hepatocellular, cholestatic, or a mixed pattern of liver damage. While diagnosis is often clinical, a liver biopsy remains the gold standard for definitive diagnosis. In the presented case, thorough investigation excluded other causes, and a clear temporal relationship between losartan initiation and liver enzyme normalization upon discontinuation supported the diagnosis [9].

Pharmacokinetically, losartan has an oral bioavailability of approximately 33%, undergoing significant first-pass metabolism. It is converted into its active metabolite by cytochrome P450 enzymes, mainly CYP2C9 and CYP3A4. The drug is excreted through feces and urine, with a plasma half-life of about two hours, whereas its active metabolites exhibit a terminal half-life of six to nine hours [1].

The diagnostic workup for suspected DILI includes liver function tests measuring alanine transaminase (ALT), aspartate transaminase (AST), alkaline phosphatase (ALP), gamma-glutamyl transpeptidase (GGT), and bilirubin levels (both total and direct). The injury is categorized based on these markers: hepatocellular injury features predominant aminotransferase elevations, while cholestatic injury is marked by elevated ALP levels. A mixed injury pattern involves features of both. The “R ratio,” defined as the ratio of ALT to ALP relative to their upper limits of normal (ULN), helps classify liver injury: a ratio >5 indicates hepatocellular damage, <2 suggests cholestatic injury, and a ratio between 2 and 5 reflects a mixed pattern. Timing is essential, as liver biopsies may show hepatocellular predominance early and cholestatic changes later [6].

Losartan-induced liver injury is exceedingly rare, affecting fewer than 0.1% of patients, and typically presents within one to eight weeks after initiation. Hepatocellular injury is the most common pattern observed, although cholestatic and mixed patterns have also been reported [10]. To date, only eight cases of losartan-induced toxic hepatitis have been documented, including the current case. In two of these, transaminase levels were reported to be 75 to 100 times above the upper normal limit [10]. Prompt discontinuation of the offending agent is the mainstay of management. In severe cases, particularly those with markedly elevated aminotransferases or signs of liver failure, treatment with N-acetylcysteine may be considered. Most patients exhibit improvement in liver enzymes within a few days; however, a small subset may progress to liver failure requiring transplantation. Re-challenging patients with the same drug is strongly discouraged due to the risk of a more severe reaction.

This case reinforces the importance of recognizing losartan as a potential cause of significant liver injury, especially in patients who develop unexplained hepatic symptoms shortly after beginning treatment. Early identification and withdrawal of the offending drug are crucial to preventing irreversible hepatic damage.

## Conclusions

Losartan-induced severe hepatic injury and sub-fulminant hepatitis are rare but potentially life-threatening adverse effects of this commonly prescribed medication. This case illustrates that, despite prompt discontinuation of the drug, rapid progression to acute liver failure necessitating transplantation can occur. Clinicians should maintain a high index of suspicion for losartan-associated hepatotoxicity in patients who develop unexplained jaundice, fatigue, abdominal pain, or elevated liver enzymes after starting the medication. Early recognition, immediate drug withdrawal, and timely referral to a transplant center are critical in managing severe cases. Further research is warranted to better understand the underlying mechanisms, identify risk factors, and establish evidence-based guidelines for monitoring and management of this uncommon complication.
